# Detection of functional deterioration in glaucoma by trend analysis using comprehensive overlapping clusters of locations

**DOI:** 10.1038/s41598-020-75619-z

**Published:** 2020-10-28

**Authors:** Stuart K. Gardiner, Steven L. Mansberger

**Affiliations:** Devers Eye Institute, Legacy Research Institute, 1225 NE 2nd Ave, Portland, OR 97232 USA

**Keywords:** Translational research, Diagnostic markers, Ocular hypertension, Optic nerve diseases, Vision disorders

## Abstract

Detecting rapid visual field deterioration is crucial for individuals with glaucoma. Cluster trend analysis detects visual field deterioration with higher sensitivity than global analyses by using predefined non-overlapping subsets of visual field locations. However, it may miss small defects that straddle cluster borders. This study introduces a comprehensive set of overlapping clusters, and assesses whether this further improves progression detection. Clusters were defined as locations from where ganglion cell axons enter the optic nerve head within a θ° wide sector, centered at 1º intervals, for various θ. Deterioration in eyes with or at risk of glaucomatous visual field loss was “detected” if ≥ *N*_*θ*_ clusters had deteriorated with p < p_Cluster_, chosen empirically to give 95% specificity based on permuting the series. *N*_*θ*_ was chosen to minimize the time to detect subsequently-confirmed deterioration in ≥ 1/3rd of eyes. Times to detect deterioration were compared using Cox survival models. Biannual series were available for 422 eyes of 214 participants. Predefined non-overlapping clusters detected subsequently-confirmed change in ≥ 1/3rd of eyes in 3.41 years (95% confidence interval 2.75–5.48 years). After equalizing specificity, no criteria based on comprehensive overlapping clusters detected deterioration significantly sooner. The quickest was 3.13 years (2.69–4.65) for θ° = 20° and *N*_*θ*_ = 25, but the comparison with non-overlapping clusters had p = 0.672. Any improvement in sensitivity for detecting deterioration when using a comprehensive set of overlapping clusters was negated by the need to maintain equal specificity. The existing cluster trend analysis using predefined non-overlapping clusters provides a useful tool for monitoring visual field progression.

## Introduction

Patients with glaucoma commonly undergo both functional and structural diagnostic testing as part of their standard clinical care. Functional testing usually takes the form of standard automated perimetry, where the test subject is presented with a series of visual stimuli of different contrast and location, and is asked to respond by pressing a button whenever stimuli are seen^1,2^. Since damage caused by glaucoma is currently irreversible, the aim of this testing is to determine how quickly damage is progressing, so that the clinician can decide whether the current management of the patient’s disease needs to be adjusted to prevent blindness within their lifetime^3,4^. However, glaucoma typically results in localized functional defects, rather than affecting all areas of the visual field equally. Thus, a global average of defect depth is insensitive to changes, with the true progression signal being swamped by considerable test–retest variability^5^. Yet, that same variability also means that estimates of contrast sensitivity at a single location are so variable that it takes several years to determine whether apparent change is real, or even statistically significant^6^. The ideal way to analyze results from clinical perimetry needs to find a balance between the two competing priorities of identifying small localized defects, while retaining the lower variability achieved from averaging information from several locations.

The key to optimizing this balance lies in the fact that the localized functional defects tend to follow specific patterns. Glaucomatous damage to retinal ganglion cells is generally assumed to occur at or near the optic nerve head (ONH)^7^; and so cells whose axons enter the ONH in close proximity to each other are more likely to become damaged at the same time^8,9^. Thus, averaging contrast sensitivity within arcuate clusters based on topographic structure–function mapping has been hypothesized as maximizing the signal-to-noise ratio and thus optimizing monitoring of the rate of disease progression. Specifically, visual field testing is most commonly performed at a regular grid of locations known as the 24–2 test pattern. Axons emanating from retinal ganglion cells whose soma (and consequent receptive field) are found at each of these retinal locations have been traced to determine their angle of entry into the ONH^10–12^. If two or more locations correspond with axons that enter the ONH in close proximity, then sensitivities at those locations are more likely to deteriorate at approximately the same rate^9^.

This principle has been applied clinically in the cluster trend analysis within the EyeSuite software developed for the Octopus perimeter (Haag-Streit Inc., Bern, Switzerland)^13^. Pointwise total deviation values (the difference in contrast sensitivity from age-matched normal) are averaged within each of ten predefined non-overlapping clusters of locations. If this average significantly deteriorates over time, then the cluster is flagged as deteriorating^13,14^. We previously showed that this approach detects glaucomatous progression sooner than global analyses, and has a higher probability than pointwise analyses that any detected deterioration will be subsequently confirmed upon further testing^15^.

However, there are two potentially sub-optimal aspects of using ten non-overlapping clusters in this way. Firstly, localized defects are not anatomically constrained to these predefined clusters, but could straddle the border between two of the clusters^15^. Secondly, the topographic structure–function relation varies between eyes due to the individualized anatomy, including physiologic variations in the exact position of the ONH relative to the visual field test locations^16,17^. Thus, some localized defects may be underestimated if they do not correspond perfectly with any single cluster, especially if they affect only half or fewer of the cluster’s locations.

Recently we took the first step towards assessing the clinical impact of these sub-optimal aspects of cluster analysis, by adding an additional set of eleven overlapping clusters to the original ten non-overlapping clusters from the EyeSuite software. However, using these 21 overlapping clusters did not significantly reduce the time to detect deterioration (for equal specificity) when compared to only using the original ten predefined non-overlapping clusters^18^. While that study represented important progress, it still relied on using the same relatively small set of fixed clusters for every eye. We therefore wanted to explore whether a more comprehensive set of overlapping clusters would provide optimal performance when compared to the original ten-cluster approach.

In this study, we describe a method to detect deterioration using comprehensive overlapping clusters of visual field locations. This method looks for deterioration among a greater number of clusters, and could therefore reduce specificity. We therefore use a permutation analysis technique^19^ to determine whether the hypothesized increase in sensitivity outweighs the potential reduction in specificity. If successful, use of a comprehensive set of overlapping clusters could improve the ability of both clinicians and researchers to rapidly and reliably detect visual field deterioration.

## Results

Series of at least five reliable visual fields were available for 422 eyes of 214 participants. 146 of those eyes (35%) had an abnormal result on the first visual field in their series, defined as either abnormal Pattern Standard Deviation (p < 5%) or a Glaucoma Hemifield Test result of either “Abnormal” or “Borderline”. 163 eyes (39%) were recorded as having been prescribed IOP-lowering medications. Table 1 summarizes other characteristics of the cohort.

**Table 1:** Characteristics of the dataset used.

	Mean	Standard deviation	Range	Interquartile range
Series length (number of visits)	14.0	4.7	5 to 23	10 to 18
Series length (years)	7.8	2.2	1.9 to 10.4	6.0 to 9.6
Age at start of series (years)	63.8	10.9	33.7 to 86.7	56.7 to 71.3
Initial mean deviation (dB)	−0.84	2.8	− 17.7 to + 3.0	− 1.4 to + 0.8
Final mean deviation (dB)	−1.71	4.0	− 26.3 to + 3.0	− 2.7 to + 0.8
Initial pattern standard deviation (dB)	2.45	2.5	0.9 to 15.7	1.4 to 2.1
Final pattern standard deviation (dB)	3.27	3.3	1.0 to 15.5	1.5 to 3.1
Rate of change of mean deviation (dB/year)	−0.13	0.3	− 1.7 to + 0.8	− 0.2 to + 0.0

Table 2 shows the time taken to detect deterioration, and the time to detect confirmed deterioration (i.e. the series was still “deteriorating” after the inclusion of the next test date in the analysis), in at least one third of the cohort (i.e. the lower tertile of survival times) using a Kaplan–Meier survival model, based on a range of different criteria. Since testing was conducted as close as possible to biannually, and only series of length ≥ 5 visits were analyzed, the first possible date at which deterioration could be detected was approximately 2 years. All the criteria based on comprehensive overlapping clusters detected confirmed deterioration significantly sooner than MD. However, none of them detected confirmed deterioration significantly sooner using the ten predefined non-overlapping clusters from the EyeSuite software.

**Table 2:** Comparison of the criteria for detection deterioration.

Criterion definition	Optimal number of clusters, *N*_*θ*_	Years to detect deterioration in ≥ 33% of eyes (95% confidence interval)	Years to detect confirmed deterioration in ≥ 33% of eyes (95% confidence interval)	Probability deterioration is confirmed on next visit	Comparison against mean deviation	Comparison against predefined clusters
Mean deviation	1	4.51 (3.28–∞)	7.22 (3.41–∞)	92.2%		
Predefined clusters	1	3.28 (2.73–5.06)	3.41 (2.75–5.48)	94.4%	< 0.001	
θ = 10º clusters	11	3.07 (2.72–4.17)	3.30 (2.74–5.90)	92.4%	< 0.001	0.806
θ = 20º clusters	25	3.03 (2.69–4.16)	3.13 (2.69–4.65)	93.0%	< 0.001	0.672
θ = 30º clusters	25	3.12 (2.72–4.17)	3.23 (2.73–4.51)	95.1%	< 0.001	0.654
θ = 45º clusters	37	3.24 (2.74–4.48)	3.32 (2.75–9.10)	93.3%	< 0.001	0.341
θ = 60º clusters	47	3.07 (2.72–4.45)	3.30 (2.74–9.10)	91.8%	< 0.001	0.356
θ = 90º clusters	34	3.28 (2.74–4.45)	3.41 (2.77–∞)	91.1%	0.001	0.194
θ = 10º unique clusters	4	3.07 (2.72–4.20)	3.41 (2.76–∞)	86.7%	0.002	0.265
θ = 20º unique clusters	5	3.28 (2.75–4.20)	3.37 (2.77–5.35)	93.4%	< 0.001	0.685
θ = 30º unique clusters	6	3.14 (2.75–4.48)	3.41 (2.81–∞)	89.0%	0.002	0.067
θ = 45º unique clusters	8	3.14 (2.73–4.47)	3.58 (2.92–∞)	87.6%	0.002	0.076
θ = 60º unique clusters	10	3.14 (2.74–4.50)	3.30 (2.76–6.98)	91.8%	< 0.001	0.482
θ = 90º unique clusters	17	3.28 (2.75–5.34)	3.43 (2.77–∞)	90.3%	0.003	0.076

For a given cluster width θ° (left column), deterioration was “detected” on the first visit at which N_θ_ clusters were deteriorating with p < p_Overall_, with this cutoff chosen to give exactly 95% specificity as detailed in the Methods. For the first set of results (labelled “θ = 10° clusters” etc.), one cluster was defined centered at each angle around the optic nerve head in 1º increments, subject to the cluster containing at least two visual field locations. For the second set of results (labelled “unique clusters”), any clusters that contained exactly the same set of field locations were only counted once. The number of clusters N_θ_ was chosen to minimize time to detect confirmed deterioration, and is shown in the second column. The next two columns give the time to detect deterioration, or subsequently-confirmed deterioration, respectively, with 95% confidence intervals. A value of ∞ indicates that too few eyes met the criterion by the end of their available series of data for this to be calculated. The next column shows the probability that deterioration was subsequently confirmed, i.e. an eye that met the criterion for deterioration still met the criterion after the addition of the next visual field in the series. The final columns show p-values for whether the criterion detected confirmed deterioration significantly sooner than using Mean Deviation, or than using the ten predefined non-overlapping clusters of locations.

The cluster criterion for cluster width θ° could be considered optimal for detecting defects that cover exactly θ° at the optic nerve head, but sub-optimal for defects of other widths. To test this, the above analysis was repeated using all available clusters of width either 10° or 30°, rather than just one width. However, this still did not detect subsequently-confirmed deterioration significantly sooner than using the ten predefined non-overlapping clusters (p = 0.497) after equalizing specificity. Neither did a similar analysis using clusters of width either 10º or 60º (p = 0.464). Using clusters of width either 30º or 60º actually detected subsequently-confirmed deterioration slightly slower than the predefined non-overlapping clusters, with p = 0.009.

**Figure 1:**
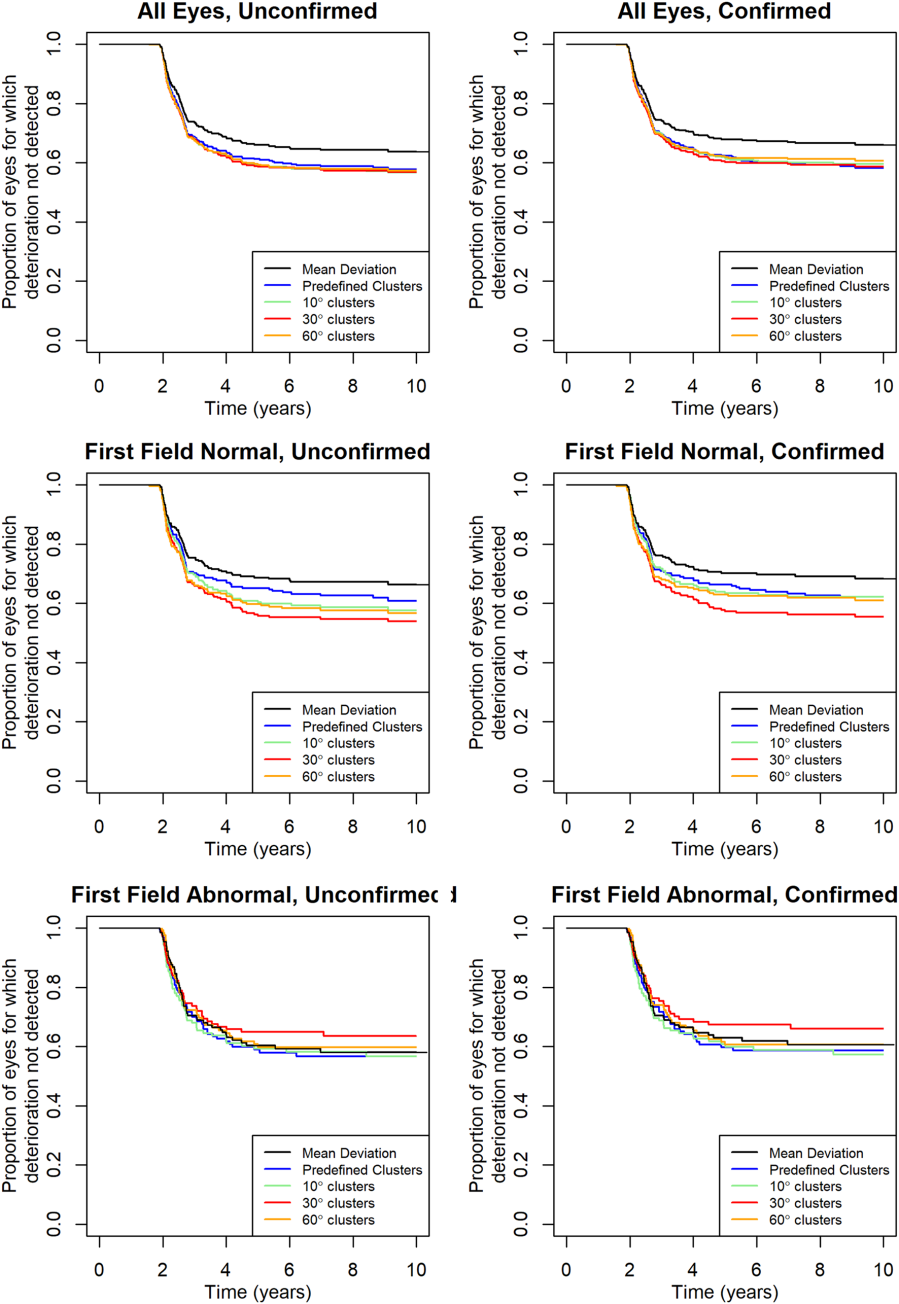
Kaplan–Meier survival plots showing the time until deterioration (left) and subsequently-confirmed deterioration (right) was detected by a selection of criteria. Plots in the top row include all 422 eyes; plots in the second row include only the 276 eyes with normal visual field at baseline; and plots in the third row include only the 146 eyes with abnormal visual field at baseline.

Table 3 shows the time to detect confirmed deterioration, and comparison against the ten predefined non-overlapping clusters, for two subsets of the cohort: 276 eyes without and 146 eyes with an abnormal visual field at the start of their series. Only 32% of eyes without an initially abnormal field showed confirmed deterioration by MD before the end of their series, hence the estimated time for 1/3rd of eyes to show confirmed deterioration in Table 3 is infinite. Unsurprisingly, deterioration was detected sooner in eyes that were abnormal at baseline, as they are more likely to progress rapidly^20^. However, while the time to detect deterioration in 1/3rd of eyes appeared shorter using some of the comprehensive overlapping cluster criteria than using predefined non-overlapping clusters, these differences were never statistically significant. Figure 1 shows Kaplan–Meier survival plots for a selection of criteria, using the optimal number of clusters for detecting confirmed deterioration in each case.

**Table 3:** Comparison of the criteria for detection deterioration as in Table 2, but differentiated into eyes with and without an abnormal visual field at baseline.

Criterion definition	Normal visual field at baseline		Abnormal visual field at baseline	
	Years to detect confirmed deterioration in ≥ 33% of eyes (95% confidence interval)	Comparison against predefined clusters	Years to detect confirmed deterioration in ≥ 33% of eyes (95% confidence interval)	Comparison against predefined clusters
Mean deviation	∞ (3.85–∞)		3.55 (2.65–∞)	
Predefined clusters	3.30 (2.72–9.10)		3.28 (2.65–6.98)	
θ = 10º clusters	3.41 (2.72–∞)	0.442	3.07 (2.63–5.90)	0.740
θ = 20º clusters	3.32 (2.69–∞)	0.993	2.77 (2.61–6.98)	0.731
θ = 30º clusters	3.31 (2.73–∞)	0.144	2.77 (2.61–6.98)	0.085
θ = 45º clusters	3.76 (2.81–∞)	0.985	2.75 (2.60–5.53)	0.237
θ = 60º clusters	3.58 (2.73–∞)	0.771	2.77 (2.63–∞)	0.338
θ = 90º clusters	4.01 (2.75–∞)	0.968	3.07 (2.65–6.98)	0.811
θ = 10º unique clusters	3.58 (2.76–∞)	0.700	3.28 (2.65–∞)	0.329
θ = 20º unique clusters	3.61 (2.76–∞)	0.907	3.07 (2.65–6.98)	0.668
θ = 30º unique clusters	3.76 (2.81–∞)	0.907	3.28 (2.65–∞)	0.536
θ = 45º unique clusters	4.17 (2.96–∞)	0.478	3.28 (2.65–∞)	0.825
θ = 60º unique clusters	3.99 (2.81–∞)	0.609	2.77 (2.61–4.64)	0.907
θ = 90º unique clusters	4.16 (2.76–∞)	0.937	3.07 (2.63–8.64)	0.295

For each subset, the two columns give the time to detect subsequently-confirmed deterioration, with 95% confidence interval in parentheses; and the p-value for whether the criterion detected confirmed deterioration significantly sooner than when using ten predefined non-overlapping clusters of locations. A value of ∞ indicates that too few eyes met the criterion by the end of their available series of data for this to be calculated.

## Discussion

Previously, we have shown that cluster trend analysis, assessing the significance of the rate of change within each of ten predefined non-overlapping clusters of test locations, provided a useful clinical tool for assessing glaucomatous progression. It detected deterioration sooner than using global metrics such as MD; and any changes that were detected were more likely to be confirmed upon subsequent retesting than when using pointwise analyses. It thus provides a useful compromise between these two competing priorities. However, we hypothesized that it could be further improved, since it could ‘miss’ defects that straddle cluster borders^9^, and/or are in eyes whose anatomy dictates a different expected topographic relation between ONH damage and visual field defect^17^. In this study, we describe a novel method to avoid those problems using an objectively-chosen set of comprehensive overlapping clusters of visual field locations. Our new technique aims to ensure that if there is a nerve fiber layer defect of width θ° at the ONH, then there will be at least one of the visual field clusters based on θ° width that exactly corresponds with this defect. However, increasing the number of possible clusters in this way causes problems with multiple comparisons, and so the criterion for deterioration has to be adjusted accordingly to achieve the same overall specificity. After making these adjustments to equalize specificity, the new technique did not detect deterioration significantly sooner than the simpler alternative of using the ten predefined non-overlapping clusters as implemented in the current clinical software^13–15^.

The strength of cluster trend analysis, compared with global or pointwise analyses, is that it better reflects the typical glaucomatous disease process which causes arcuate field loss due to the spatial arrangement of nerve fiber axon bundles in the retina^9^. Generalized loss, affecting the entire visual field approximately equally, would presumably be better detected by global indices. Such loss does appear in glaucoma^21–24^, but is of relatively small magnitude compared with localized scotoma; thus deterioration can be detected sooner when focusing on localized changes^15^. Defects that cover only a single visual field location in the 24–2 test pattern can also occur, especially in the central region of the field^25^; but apparent loss at a single location is often just due to variability and so may not be subsequently confirmed^26–28^. The ideal balance between these issues will depend on the clinical situation. In particular, a small localized defect may be ‘trusted’ more if there is a structural defect at the corresponding location in the nerve fiber layer. No single analytic tool is optimal in all clinical scenarios. However, there is now compelling evidence that cluster-based analyses should be one of the set of tools made available to clinicians; and that perceived flaws such as its reliance on a limited predefined set of clusters do not significantly reduce its usefulness.

Although testing was performed using clinical instruments and protocols, there are still potentially important differences between this study and typical clinical practice. Eyes were tested once every six months, or as close as could be scheduled, whereas it would be more common clinically to test people more frequently if rapid progression were suspected^29^. Study participants are also highly experienced with automated perimetry, and there may be greater benefits of averaging information from larger numbers of locations in less-experienced patients who typically have higher test–retest variability^30,31^.

The majority of the cohort had either no visual field loss or early loss; even at the end of the series the average MD was − 1.7 dB. Both eyes were tested, even if clinically only one eye would be considered glaucomatous. Eyes with glaucoma were being managed clinically to slow their rates of progression, and given that these participants were motivated enough to participate in a long-term study, they could be hypothesized to have greater compliance with their prescribed medications than a more general population. For all these reasons, we would not expect many eyes to show rapid progression^20,32^. There are no obvious reasons to suppose that comparisons between criteria would be different in a cohort of eyes undergoing more rapid progression, and indeed an eye that is progressing sufficiently rapidly would be detected by any of the tested criteria; but such a differential effect cannot be ruled out.

The analysis technique used in this study relies on ordinary least squares regression for each series. However, the overall p-values are derived empirically by comparison against the permutation distribution. This reduces caveats concerning the validity of the underlying assumptions of the analysis, in particular with regard to normality of the residuals. Some eyes could be more variable than others, both due to individual factors^33^ and due to the increase in variability with disease severity^34^. A mixed effects model would typically assume homogeneity of the residuals, but permutation analysis only requires the much weaker assumption of homogeneity within the series for an individual eye. Another caveat with the analysis is that no adjustment was made for multiple comparisons using different sector widths θ°, and so the probability of finding a statistically significant difference was inflated; yet even without such adjustment, no such significant differences were found.

In conclusion, we found that although cluster trend analysis as implemented in the clinical EyeSuite software uses only ten predefined non-overlapping clusters of locations instead of a more comprehensive evaluation of possible clusters, this does not significantly delay the detection of visual field deterioration. Any benefit from identifying more defects using a more comprehensive set of clusters was negated by the adjustments to the criteria needed to maintain specificity. Cluster trend analysis provides a useful tool for monitoring deterioration in glaucomatous visual fields.

## Methods

### Participants

The same data were used as in our previous study^18^. This was a retrospective analysis of data taken from the ongoing Portland Progression Project (P3), conducted at Legacy Devers Eye Institute, in which participants undergo a range of structural and functional testing once every six months. Inclusion criteria were a diagnosis of primary open-angle glaucoma and/or likelihood of developing glaucomatous damage, as determined subjectively by each participant’s physician, in order to reflect current clinical practice. Exclusion criteria were an inability to perform reliable visual field testing, best-corrected visual acuity at baseline worse than 20/40, significant cataract or media opacities likely to significantly increase light scatter, or other conditions or medications that affect the visual field. Standard automated perimetry was performed using a Humphrey Field Analyzer HFAIIi perimeter, with the 24–2 test pattern, a size III white-on-white stimulus, and the SITA Standard algorithm^35^. Eyes were included in the analyses for this study if they had at least five reliable tests, defined as ≤ 15% false positives and ≤ 33% fixation losses. All protocols were approved and monitored by the Legacy Health Institutional Review Board, and adhered to the Health Insurance Portability and Accountability Act of 1996 and the tenets of the Declaration of Helsinki. All participants provided written informed consent once all of the risks and benefits of participation were explained to them.

### Analysis—definition of comprehensive overlapping clusters

The comprehensive clustering system used in this study relies on the structure–function map published by Garway-Heath et al.^10^ In that study, axon bundles were manually traced on nerve fiber layer photographs from each test location to the point at which they entered the ONH. This gave an average angle of entry for axons corresponding with each location in the 24–2 test pattern. A “cluster” of visual field locations in this study was defined as the set of locations whose corresponding axons enter the ONH between α − (θ/2)º and α + (θ/2)º, for a chosen center αº and width θº.

**Figure 2:**
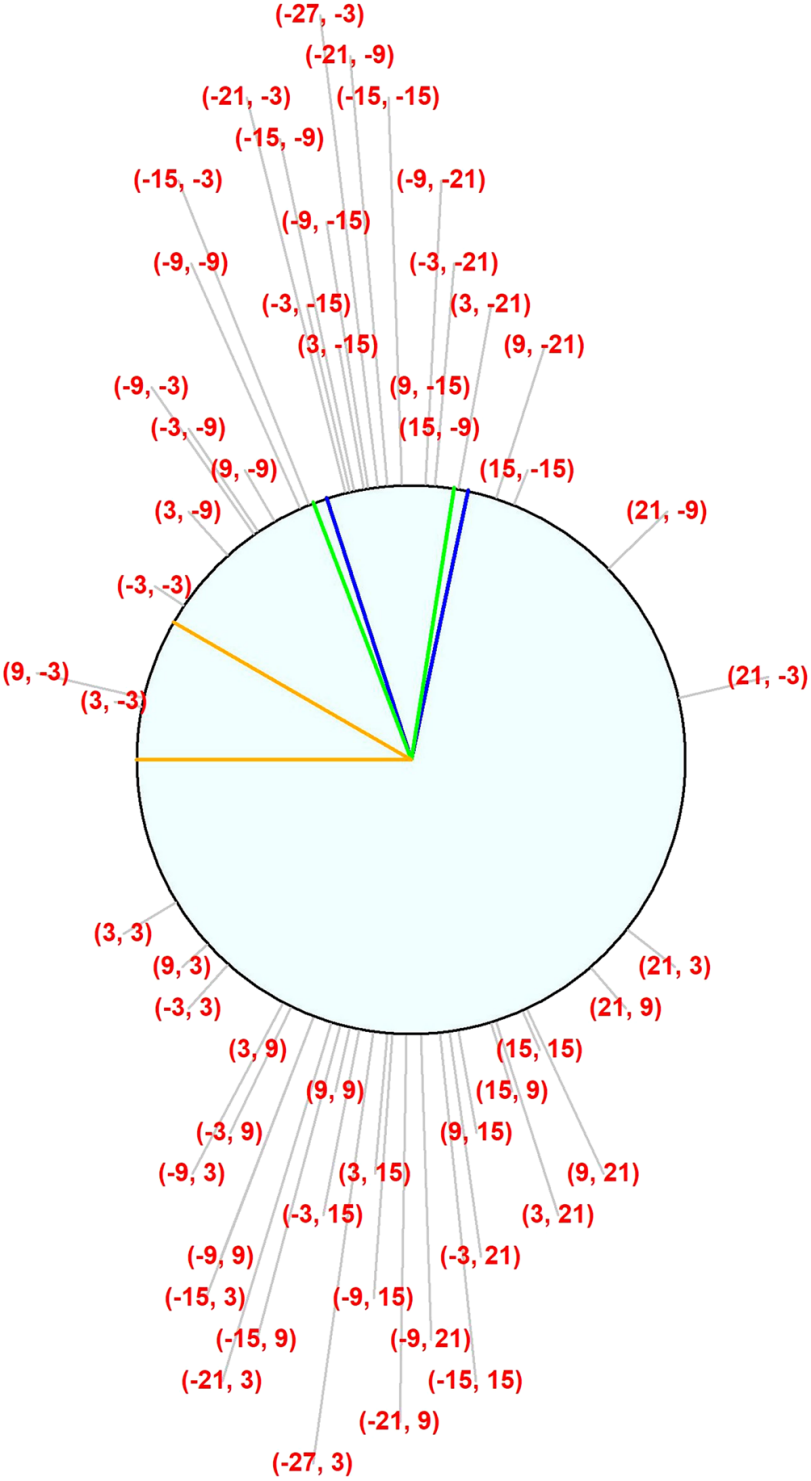
Illustration of the clustering system used. Numbers in red give the coordinates (in degrees) of each visual field location for a right eye. Gray lines show the angle at which the corresponding axons enter the optic nerve head. The 30º sector shown by the green lines contains 12 visual field locations. The 30º sector shown by the blue lines contains the same 12 visual field locations, plus one more location (3, − 21). The 30º sector shown by the orange lines only contains 2 visual field locations, (9, − 3) and (3, − 3).

The exact topographic relation varies; the reported standard deviation of these traced angles between eyes was 7.2º^10^. A structural defect in the nerve fiber layer could also be centered at any angle around the ONH. Therefore, one cluster was formed for every possible center αº, at 1º intervals. Thus, any nerve fiber layer defect of width θº can be assumed to exactly correspond with one of these clusters. This remains true even if the rotational error from the average eye is not constant around the disc, for example due to inter-individual differences in axial length^16^. To avoid excessive variability, only clusters containing at least two visual field locations were considered. Hence, there were fewer than 360 clusters for any given width θ°, with the actual number increasing with θ°. Figure 2 shows the average angle of entry to the ONH for axons corresponding to each visual field location in a right eye, together with three of the clusters based on a width of θ° = 30°. Note that several clusters centered at neighboring angles α° could consist of the same set of visual field locations. For the primary analysis, these were considered as separate clusters. A secondary analysis was performed using only “unique clusters”, in which the same set of visual field locations is never included more than once.

### Analysis—detecting deterioration

The total deviation (on the native decibel scale) was averaged across locations within each eligible cluster of width θ°^15^. Total deviation values for each location were used instead of raw sensitivities to account for the effect of normal aging; therefore in the absence of disease progression each cluster would be expected to show zero change over time. We have previously shown that “censoring” sensitivities below 15 dB and setting them equal to 15 dB improves reliability and hence the ability to detect change. Thus, total deviation values for any such locations were set to equal the total deviation value for a sensitivity of 15 dB^36,37^. All analyses were performed using the R statistical programming language (Version 4.0.0)^38^.

Seeking deterioration in any one of multiple clusters would be expected to increase sensitivity, but at the cost of reduced specificity. In order to fairly compare criteria given the competing demands of sensitivity vs. specificity, we sought to establish criteria for whether an eye is deteriorating with exactly 95% specificity, based on the previously published Permutation Analyses of Pointwise Linear Regression (PoPLR) approach^19^.

For each cluster of locations, *p*_*Cluster*_ was defined as the p-value from an ordinary least squares regression of the average total deviation against time. For a given number of clusters *N*, *p*_*Overall*_ was defined as the *N*th smallest of these *p*_*Cluster*_ values. A permutation distribution for *p*_*Overall*_ was derived by repeatedly reordering visual fields 1 − *V*, but retaining the original test dates. For *V* = 5, this was done for all 120 possible reorderings of the 5 visits; for *V* > 5, 475 randomly-chosen reorderings were used, to avoid excessive computation time (475 reorderings allows a specificity of 95% to be calculated with a confidence interval of ± 1% based on a binomial distribution). “Deterioration” was detected on the first visit *V* for which *p*_*Overall*_ was below the 5^th^ percentile of the permutation distribution. Thus, a group of criteria are derived each with specificity exactly equal to 95%, but with different numbers of clusters *N*, and different cluster widths θ°.

### Analysis—confirmation of deterioration

“Confirmed deterioration” was detected on the first visit *V* at which the *N*th smallest observed p-value was below the 5th percentile of the *N*th smallest p-values from all reorderings, for both the series 1 through *V* and the series 1 through (*V* + 1). The date of detection is defined as being visit *V*, not visit *V* + 1. It was not necessary for the *N*th smallest p-value to come from the same sector for both time points. The probability that “confirmed deterioration” was detected on the same date that “deterioration” was detected can then be taken as a metric of the robustness of a particular analysis^26,39^.

### Analysis—comparison of criteria

For each width θ°, the optimal number of clusters *N*_*θ*_ was chosen as the criteria that detected subsequently-confirmed deterioration in ≥ 33% of eyes soonest, based on a Kaplan–Meier survival analysis. In the event of a tie, the smallest such number *N* was used as *N*_*θ*_ thereafter. The optimal criteria using *N*_*θ*_ clusters of width θ° were compared against each other; and also against using *N* of the ten predefined non-overlapping clusters from the EyeSuite software in the same manner, or using Mean Deviation (effectively the same analysis as above but with *N* = 1 and θ = 360°).

As in our previous study^18^, for each criterion, Kaplan–Meier survival analysis was used to determine the time taken until ≥ 33% of eyes had shown “deterioration” or “confirmed deterioration”. 95% confidence intervals for these times were found using standard errors based on Greenwood’s formula^40^. Survival curves were compared using a stratified Cox proportional hazards model^41^, with strata identifying fellow eyes of the same individual^42^. Sub-analyses were performed within the subset of eyes that were abnormal at the start of their series, defined as either abnormal Pattern Standard Deviation (p < 5%) or a Glaucoma Hemifield Test result of either “Abnormal” or “Borderline”; and within the subset of eyes that did not meet those criteria and so would be considered normal at the start of their series.

## Data Availability

The datasets analyzed during the current study are available from the corresponding author on reasonable request.
